# Factors associated with sufficient knowledge of antibiotics and antimicrobial resistance in the Japanese general population

**DOI:** 10.1038/s41598-020-60444-1

**Published:** 2020-02-26

**Authors:** Shinya Tsuzuki, Niina Fujitsuka, Keisuke Horiuchi, Shinpei Ijichi, Yoshiaki Gu, Yumiko Fujitomo, Rie Takahashi, Norio Ohmagari

**Affiliations:** 1 AMR Clinical Reference Center, National Center for Global Health and Medicine, Tokyo, Japan; 2 Faculty of Medicine and Health Sciences, University of Antwerp, Antwerp, Belgium; 3 NTT Data, Tokyo, Japan; 4 DataRobot Japan, Tokyo, Japan; 5 Disease Control and Prevention Center, National Center for Global Health and Medicine, Tokyo, Japan

**Keywords:** Infectious diseases, Public health

## Abstract

We conducted two online surveys about antibiotics targeted at the Japanese general population in March 2017 and February 2018. In total, 6,982 participants completed the questionnaire. Factors associated with knowledge of antibiotics, knowledge of antimicrobial resistance (AMR) and appropriate behavioural changes were evaluated by a machine learning approach using DataRobot. Factors strongly associated with three dependent variables in the model were extracted based on permuation importance. We found that the strongest determinant of knowledge of antibiotics and AMR was education level. Knowledge of antibiotics was strongly associated with the frequency of internet use. Exposure to primary information was associated with motivation for appropriate behavioural changes. Improving the availability of primary information would be a beneficial intervention. Individuals lacking higher education and without opportunities to obtain primary information should be considered a target population for effective interventions.

## Introduction

Antimicrobial resistance (AMR) is currently one of the greatest threats to global health^1,2^. A year after the publication of the Global Action Plan on Antimicrobial Resistance in 2015^1^, the Japanese government established a National Action Plan^3^. According to this plan, comprehensive countermeasures against AMR are needed, including educational activities for the general population.

The National Center for Global Health and Medicine in Japan established the AMR Clinical Reference Center as the main hub of information, educational activities and research related to AMR in Japan.

Some studies have evaluated the general population’s knowledge of antibiotics and AMR and related behaviours^4–10^. A systematic review has shown that the general population typically understands that excessive or unnecessary antibiotic use and the incompletion of antibiotic courses cause antibiotic resistance^6^. However, Kamata and colleagues have reported that a larger proportion of the Japanese general population demonstrates insufficient knowledge and inappropriate behaviours towards antibiotics and AMR than the general populations of European countries^11^. Therefore, providing sufficient information about AMR to the Japanese general population might be an important AMR countermeasure in the implementation of national health policy.

However, in the qualitative analysis by Kamata and colleagues, the factors associated with sufficient knowledge and appropriate behavioural changes towards antibiotics and AMR in Japan were not identified. An understanding of the precise determinants of sufficient knowledge and motivation for appropriate behavioural changes can provide a basis for the development of more effective interventions. In this study, we used a questionnaire-based approach to identify the key determinants of knowledge and behaviours related to antibiotics and AMR in Japan.

## Results

In total, 1,351 (19.3%) and 824 (11.8%) of participants demonstrated sufficient knowledge of antibiotics and AMR, respectively, and 447 (25.5%) showed motivation to make appropriate behavioural changes. The details and results of questions related to antibiotics, AMR, and beliefs and behaviours are available in Tables 1–3.

**Table 1:** Responses to questions about antibiotics.

	2017	2018	Total
**Any antibiotic use within 1 year**			
Yes	1,566 (46.2)	1,737 (48.4)	3,303 (47.3)
No	1,824 (53.8)	1,855 (51.6)	3,679 (52.7)
**1. Antibiotics kill viruses**			
True (incorrect)	1,587 (46.8)	1,675 (46.6)	3,262 (46.7)
False (correct)	741 (21.9)	730 (20.3)	1,471 (21.1)
Do not know	1,062 (31.3)	1,187 (33.0)	2,249 (32.2)
**2. Antibiotics are effective for the common cold and influenza**			
True (incorrect)	1,376 (40.6)	1,572 (43.8)	2,948 (42.2)
False (correct)	835 (24.6)	794 (22.1)	1,629 (23.3)
Do not know	1,179 (34.8)	1,226 (34.1)	2,405 (34.4)
**3. Unnecessary use of antibiotics makes them ineffective**			
True (correct)	2,288 (67.5)	2,473 (68.8)	4,761 (68.2)
False (incorrect)	106 (3.1)	132 (3.7)	238 (3.4)
Do not know	996 (29.4)	987 (27.5)	1,983 (28.4)
**4. Frequent use of antibiotics often results in side-effects, such as diarrhoea**			
True (correct)	1,315 (38.8)	1,492 (41.5)	2,807 (40.2)
False (incorrect)	429 (12.7)	483 (13.4)	912 (13.1)
Do not know	1,646 (48.6)	1,617 (45.0)	3,263 (46.7)
**Has appropriate knowledge of antibiotics (3 or 4 correct answers)**			
Yes	663 (19.6)	688 (19.2)	1,351 (19.3)
No	2,727 (80.4)	2,904 (80.8)	5,631 (80.7)

Numbers in parentheses indicate percentages.

**Table 2:** Responses to questions about AMR.

	2017	2018	Total
**Have heard the term “AMR (*yakuzai taisei*** **in Japanese)”**			
Yes	1,409 (41.6)	1,534 (42.7)	2,943 (42.2)
No	1,981 (58.4)	2,058 (57.3)	4,039 (57.8)
**1 a) AMR refers to when humans become immune to antibiotics**			
True (incorrect)	1,415 (41.7)	1,522 (42.4)	2,937 (42.1)
False (correct)	331 (9.8)	314 (8.7)	645 (9.2)
Do not know	1,644 (48.5)	1,756 (48.9)	3,400 (48.7)
**1 b) AMR means bacteria that avoid being killed by antibiotics**			
True (correct)	1,467 (43.3)	1,538 (42.8)	3,005 (43.0)
False (incorrect)	151 (4.5)	167 (4.6)	318 (4.6)
Do not know	1,772 (52.3)	1,887 (52.5)	3,659 (52.4)
**2. What causes AMR? (multiple answers allowed)**			
A. Unnecessary use of antibiotics	1,246 (36.8)	1,371 (38.2)	2,617 (37.5)
B. Antibiotic abuse	1,578 (46.5)	1,744 (48.6)	3,322 (47.6)
C. Lack of countermeasures in healthcare facilities	206 (6.1)	213 (5.9)	419 (6.0)
D. Interruption of antibiotics	481 (14.2)	496 (13.8)	977 (14.0)
E. Others	51 (1.5)	34 (0.9)	85 (1.2)
F. Do not know	1,247 (36.8)	1,275 (35.5)	2,522 (36.1)
Has appropriate knowledge of AMR*			
Yes	401 (11.8)	423 (11.8)	824 (11.8)
No	2,989 (88.2)	3,169 (88.2)	6,158 (88.2)

Numbers in parentheses represent percentages. AMR; antimicrobial resistance.

*One point is assigned for correct answers to both questions 1 a) and b). Choices A, B, C, and D for question 2 are one point each (maximum four points). If F is chosen for question 2, the final score for question 2 is be 0, regardless of other choices. E does not affect the final score for question 2. The total score is the sum of scores for questions 1 and 2 (range, 0–5). Total scores of three, four, and five indicate sufficient knowledge of AMR.

**Table 3:** Responses to questions about behavioural change.

	2017	2018	Total
**Has had opportunities to obtain knowledge about AMR in the past year**			
Yes	571 (16.8)	566 (15.8)	1,137 (16.3)
Already had	869 (25.6)	956 (26.6)	1,825 (26.1)
No	1,950 (57.5)	2,070 (57.6)	4,020 (57.6)
Behavioural change after obtaining knowledge about AMR*			
Yes	212 (25.0)	235 (26.0)	447 (25.5)
No	636 (75.0)	669 (74.0)	1305 (74.5)

Numbers in parentheses represent percentages. AMR; antimicrobial resistance.

*Respondents who indicated two or more behavioural changes among those who had the opportunity to obtain knowledge about AMR. Details are available in Supplementary File.

We generated three datasets from the original data with three partition types. For over 30 models, the fit was compared for each dependent variable (knowledge of antibiotics, knowledge of AMR, and behavioural change). We obtained the model with the largest AUC value for each dependent variable in each dataset, yielding nine models for three dependent variables.

**Figure 1:**
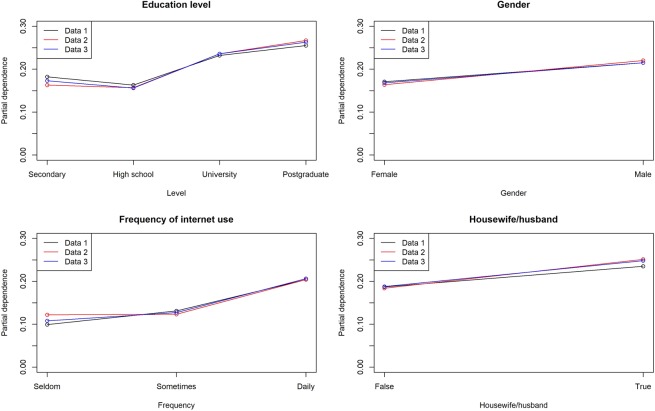
Partial dependence of variables strongly associated with having appropriate knowledge of antibiotics.

**Figure 2:**
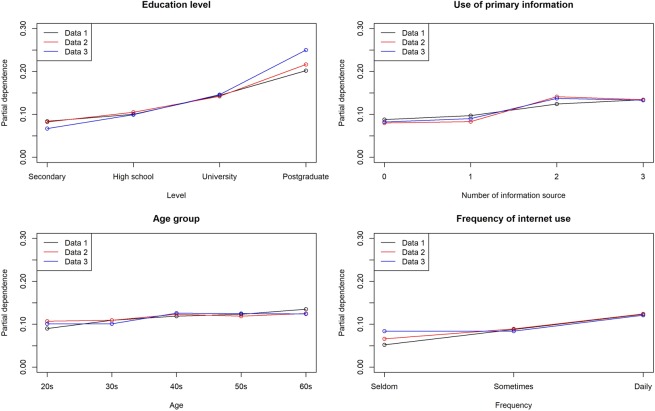
Partial dependence of variables strongly associated with having appropriate knowledge of AMR.

**Figure 3:**
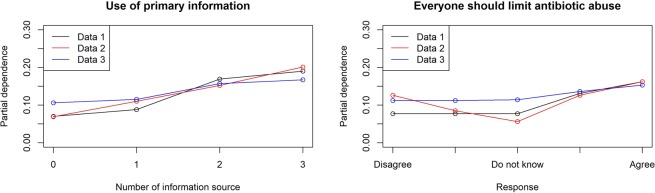
Partial dependence of variables strongly associated with behavioural changes after obtaining knowledge of AMR.

The factor that was most strongly associated with knowledge of antibiotics and AMR was education level (permutation importance were 1.0 in all datasets for both). Knowledge of antibiotics was strongly associated with the frequency of internet use (permutation importance was 0.62, 0.49, and 0.35 in each dataset). Exposure to primary information was strongly associated with motivation to make appropriate behavioural changes (permutation importance was 1.0, 0.86, and 1.0 in each dataset). The details of the models and factors strongly associated with each dependent variable are shown in Tables 4 and 5. Besides, Figures 1–3 represent each variable’s partial dependence value. Partial dependence is an indicator which represents the marginal effect of features on the predicted outcome of a machine learning model after we have averaged out the influence of all other variables.

**Table 4:** Best fitting model for each dataset.

	Data 1	Data 2	Data 3
**Has sufficient knowledge of antibiotics**			
Model	ENET blender	Regularized logistic regression	Regularized logistic regression
AUC*	0.646	0.627	0.654
AUC (Five-fold)**	0.625	0.627	0.626
**Has sufficient knowledge of AMR**			
Model	ENET blender	ENET blender	Regularized logistic regression
AUC*	0.601	0.662	0.607
AUC (Five-fold)**	0.620	0.619	0.619
**Behavioural change after obtaining knowledge of AMR**			
Model	eXtreme gradient boosted tree classifier	Regularized logistic regression	Gradient boosted tree classifier
AUC*	0.642	0.683	0.625
AUC (Five-fold)**	0.630	0.641	0.631

AMR; antimicrobial resistance, ENET; elastic-net, AUC; Area under the curve.

*AUC value from validation with the first subset among five subsets.

**AUC value from Five-fold validation with all the subsets.

**Table 5:** Common variables strongly associated* with the dependent variables in all datasets.

	Data 1	Data 2	Data 3
**Has sufficient knowledge of antibiotics**			
Education level	1.0	1.0	1.0
Male	0.46	0.58	0.42
Frequency of internet use	0.62	0.49	0.35
Housewife/husbund	0.35	0.53	0.30
**Has sufficient knowledge of AMR**			
Education level	1.0	1.0	1.0
Use of primary information	0.30	0.30	0.31
Age group	0.30	0.30	0.13
Frequency of internet use	0.28	0.28	0.10
**Behavioural change after obtaining knowledge of AMR**			
Use of primary information	1.0	0.86	1.0
Everyone should limit antibiotic abuse for the next generation	0.22	0.39	0.33

Numbers represent permutation importance. AMR; antimicrobial resistance.

*Variables ranked among the top 10 in permutation importance in all three datasets.

Details of the selected models are provided in Supplementary File S2.

## Discussion

This study provides the first evidence for an association between socioeconomic factors and sufficient knowledge of AMR in Japan. It should be noted that age is not consistently regarded as a determinant of sufficient knowledge, although several previous studies have reported that antibiotic use tends to be more frequent in older individuals than in relatively young individuals^7^. A few recent studies have reported an association between education level and knowledge of antibiotics and AMR^10^, which is consistent with our results. Therefore, targeted interventions based on education level might be more effective than existing interventions aimed at the general public as a whole. For example, educational programmes at primary schools such as e-Bug by Public Health England^12,13^ are promising.

Our results also showed that the source of information about antibiotics and AMR might play an important role in the knowledge level of the general public. In previous studies of sources of information about antibiotics and AMR^14–17^, the Internet has not been identified as an important source. These results highlight the importance of information source for the implementation of educational interventions targeting the general public.

Napolitano and colleagues pointed out that parents who do not refer to physicians as an information source tend to use antibiotics more frequently^14^. Hoffmann and colleagues reported that patients who obtain information from general practitioners are more informed about antibiotics and AMR than those who obtain information from other sources^15^. Additionally, that study showed that higher education level is more strongly associated with a higher score than information source. Those findings are compatible with our present results indicating that education level and exposure to primary information are strongly associated with the motivation for appropriate behavioural changes. These findings taken together show that exposure to primary information, especially information provided by healthcare professionals, may promote effective changes in antibiotic consumption.

However, internet use has not previously been linked to sufficient knowledge of antibiotics and AMR. One of our key findings was that frequent internet use is an independent factor for having sufficient knowledge about antibiotics; this seems to contradict previous findings that primary information is important for sufficient knowledge about AMR and appropriate behavioural changes. It is possible that the existence of antibiotics has become general knowledge and therefore the public can easily access information about antibiotics. However, the concept of AMR is relatively novel for the general public because only 3 years have passed since the National Action Plan was issued by the Japanese government. In addition, people who use the Internet frequently might have a higher sensitivity to information than those do not use the Internet and tend to search for new terms more frequently. As a result, internet-based information tends to present both advantages and disadvantages, and primary information from healthcare professionals becomes more reliable. Although further research about the effect of information source is needed, our data clearly indicate that the frequent provision of primary information would substantially improve the knowledge and behaviour of the general public.

This study has some strengths and limitations. One important strength that differentiates our analysis from previous studies is its methodology. DataRobot, which we used to conduct all analyses, can rapidly compare many model types. Accordingly, we can choose the optimal model among dozens of models and avoid arbitrariness in model selection. However, it is important to note that the AUC values of the models were relatively low, despite this strict model selection process. A larger number of variables is probably needed to explain the association of knowledge and behaviour with socioeconomic factors. In addition, the questionnaire was administered online. Therefore, respondents were internet literate and might represent a biased sample.

In conclusion, our results confirm the importance of education level in having sufficient knowledge of antibiotics and AMR. The use of primary information was strongly associated with both knowledge and behavioural changes, suggesting that the information source could be an important factor for improving the general public’s knowledge of AMR. Although our results should be interpreted with caution, they could help health policy decisionmakers conduct educational interventions directed at the general public.

## Methods

### Data source

A web-based questionnaire was developed to collect responses anonymously. When participants first visited the survey website, the policy for the use of the collected data and the protection of personal information was displayed. The details of the questionnaire are available as Supplementary File S1.

The online nationwide cross-sectional survey was conducted twice, in March 2017 and February 2018. The participants were selected among Japanese adults aged 20–69 years from a public panel in which 7.6 million people were registered by a research company (INTAGE Corporation). A total of 6,982 complete survey responses were obtained. Individuals aged 70 years or older were excluded owing to potential difficulties in responding to online surveys and to match the age criterion applied in a similar European study (20–69 years)^4^. In addition, the participants were selected to reflect the general population (based on the national population census of Japan in 2015) in terms of sex, age, place of residence and population size. Participant characteristics and their responses to other questions related to antibiotics and AMR are shown in Tables 6 and 7.

**Table 6:** Basic characteristics of survey participants.

	2017	2018	Total
**Sex**			
Male	1,736 (51.2)	1,806 (50.3)	3,542 (50.7)
Female	1,654 (48.8)	1,786 (49.7)	3,440 (49.3)
**Age group (years)**			
20–30	428 (12.6)	446 (12.4)	874 (12.5)
30–40	789 (23.3)	771 (21.5)	1,560 (22.3)
40–50	771 (22.7)	845 (23.5)	1,616 (23.1)
50–60	861 (25.4)	908 (25.3)	1,769 (25.3)
60–70	541 (16.0)	622 (17.3)	1,163 (16.7)
**Profession**			
Education	137 (4.0)	153 (4.3)	290 (4.1)
Student	102 (3.0)	108 (3.0)	210 (3.0)
Other	3,151 (93.0)	3,331 (92.7)	6,482 (92.9)
**Education level**			
Secondary	111 (3.3)	105 (2.9)	216 (3.1)
High school	1,265 (37.3)	1,374 (38.3)	2,639 (37.8)
Vocational college	774 (22.8)	771 (21.5)	1,545 (22.1)
University	1,070 (31.6)	1,170 (32.6)	2,240 (32.1)
Postgraduate	88 (2.6)	80 (2.2)	168 (2.4)
Other	82 (2.4)	92 (2.6)	174 (2.5)
**Frequency of internet use**			
Daily	2,900 (85.5)	3,094 (86.1)	5,994 (85.8)
Sometimes	357 (10.5)	394 (11.0)	751 (10.8)
Seldom	133 (3.9)	104 (2.9)	237 (3.4)
**City size**			
Metropolitan	1,014 (29.9)	1,099 (30.6)	2,113 (30.3)
Midsize city	1,439 (42.4)	1,561 (43.5)	3,000 (43.0)
Small city	683 (20.1)	687 (19.1)	1,370 (19.6)
Rural	254 (7.5)	245 (6.8)	499 (7.1)

Numbers in parentheses indicate percentages.

**Table 7:** Responses to questions about beliefs and behaviours related to antibiotics and AMR.

	2017	2018	Total
**Everyone should limit antibiotic abuse for the next generation**			
Agree	1,707 (50.4)	1,886 (52.5)	3593 (51.5)
Partially agree	1,067 (31.5)	1,126 (31.3)	2193 (31.4)
Partially disagree	77 (2.3)	75 (2.1)	152 (2.2)
Disagree	28 (0.8)	25 (0.7)	53 (0.8)
Do not know	511 (15.1)	480 (13.4)	991 (14.2)
**Source of information about antibiotics and AMR (multiple answers allowed)**			
Primary*	2,889 (85.2)	3,162 (88.0)	6,051 (86.7)
Secondary**	1,201 (35.4)	1,184 (33.0)	2,385 (34.2)
None	39 (1.2)	30 (0.8)	69 (1.0)
Do not know	228 (6.7)	199 (5.5)	427 (6.1)
**Have caught a cold and/or flu in the past 5 years**			
Yes	2,032 (59.9)	2,178 (60.6)	4,210 (60.3)
No	1,358 (40.1)	1,414 (39.4)	2,772 (39.7)
**Have quit antibiotic treatment before course completion**			
Yes	801 (23.6)	861 (24.0)	1,662 (23.8)
No	2,589 (76.4)	2731 (76.0)	5,320 (76.2)
**Keep antibiotics at home**			
Yes	396 (11.7)	426 (11.9)	822 (11.8)
No	2,994 (88.3)	3,166 (88.1)	6160 (88.2)
**Have requested physicians to prescribe antibiotics**			
Yes	345 (10.2)	430 (12.0)	775 (11.1)
No	3,045 (89.8)	3,162 (88.0)	6207 (88.9)
**Prefer physicians who prescribe antibiotics**			
Yes	1,023 (30.2)	1,196 (33.3)	2,219 (31.8)
No	2,367 (69.8)	2,396 (66.7)	4,763 (68.2)

Numbers in parentheses indicate percentages. AMR; antimicrobial resistance.

*Information provided by healthcare professionals, research institutes, and governmental organizations.

**Information provided by family, friends, private individuals, private companies, and mass media (television and journals).

To examine factors associated with sufficient knowledge of antibiotics and AMR, data from all 6,982 respondents were used. To identify factors associated with appropriate behavioural changes, data from 2,962 respondents were used. The sample sizes differed because the questions addressing behavioural changes could only be answered by those who initially answered that their behaviour had changed.

### Data preparation and pre-processing

#### Outcome

Knowledge of antibiotics was determined based on four questionnaire items. Two or fewer correct answers indicated a lack of knowledge whereas three or more correct responses indicated sufficient knowledge.

Knowledge of AMR was determined based on the results of two questions. The first question consisted of two small questions that were considered correct only if both were answered correctly. The second question asked respondents about the causes of AMR, and comprised a multiple-choice question with six options, four of which were regarded as correct statements. The other two options were “Others” and “Do not know”. The former was excluded from the calculation of the number of correct answers because the correctness depends on precise responses. If the respondent selected “Do not know”, the number of correct answers was 0, regardless of other choices. Accordingly, the total number of correct answers ranged from 0 to 5. Respondents with two or fewer correct answers were regarded as lacking knowledge about AMR whereas those with three or more correct responses were regarded as having sufficient knowledge about AMR.

As for behavioural change after obtaining knowledge about AMR, respondents who indicated two or more behavioural changes among those who had the opportunity to obtain knowledge about AMR were regarded as changed their behaviour. The details of the question about behavioural change are available in Supplementary File S1.

#### Pre-processing

Various pre-processing methods were automatically applied to the data. For categorical values, the pre-processing methods included ‘one-hot encoding’^18^ and ‘ordinary encoding’. For numerical values, ‘standardization’^19^, ‘constant splines’^20^, and ‘imputing missing values’^21^ were used.

### Validation

Data were separated into training and validation sets. Cross-validation was used for model construction and evaluation. Five-fold cross validation was used, and the partitions were determined with stratified sampling. Each of the three types of partitions were obtained with different random seeds.

As an optimization metric, logarithmic loss was used.

### Model building by machine learning

Models were created using the automated machine learning platform DataRobot. It was used to create over 40 models, including “blender models” obtained by using several machine learning algorithms. A blender model, sometimes referred to as an *ensemble model*, increases accuracy by combining the predictions of two or more models. The best model of all developed models was selected based on the largest area under the curve (AUC) value. All analyses were conducted on 4 December 2019. The details of variables included in each analysis are available in Table 8.

**Table 8:** Explanatory variables included in each analysis.

Dependent variable of each analysis	Has sufficient knowledge of antibiotics	Has sufficient knowledge of AMR	Behavioural change after obtaining knowledge of AMR
Explanatory variables common to all dependent variables	Age group		
	Sex		
	Education level		
	Frequency of internet use		
	Any antibiotic use within 1 year		
	Where you obtain antibiotics		
	Reason for taking antibiotics		
	Keep antibiotics at home		
	Have quit antibiotic treatment before course completion		
	Have used the antibiotics you keep at home		
	Have given the antibiotics you keep at home to others		
	Have caught a cold and/or flu in the past 5 years		
	Have requested physicians to prescribe antibiotics		
	Teacher		
	Student		
	Housewife/husband		
	City size		
	Life expectancy of the prefecture you live		
	Use of primary information		
Additional variables included in the third dependent variable			Prefer physicians who prescribe antibiotics
			Has had opportunities to obtain knowledge about AMR in the past year
			Everyone should limit antibiotic abuse for the next generation

AMR; antimicrobial resistance.

### Evaluation

Factors were identified based on permutation importance^22^. Permutation importance measures how much worse a model would perform if DataRobot made predictions after randomly shuffling the elements in a given column (while leaving the other columns unchanged). DataRobot normalizes the scores by setting the value of the most important column to 1.

The influence of changes in values for each factor on the outcome was also evaluated based on partial dependence^23^. A partial dependence plot was generated to show the marginal effect of features on the predicted outcome of a machine learning model.

### Sensitivity analysis

We conducted sensitivity analyses by multi-class classification approach. We classified all participants in accordance with the score each of them marked. This approach did not improve accuracy of precision substantially in view of AUC value then we deployed the original approach as our main results. The details of sensitivity analyses are available in Supplementary File S3.

### Ethics approval

This research was approved by the institutional ethical review board of the National Center for Global Health and Medicine, Tokyo, Japan and was conducted in accordance with the approved guidelines.

### Transparency declarations

The first and the corresponding author (ST) affirms that this manuscript is an honest, accurate, and transparent account of the study; that no important aspects of the study have been omitted; and that any discrepancies from the study as planned (and, if relevant, registered) have been explained.

## Supplementary information

Supplemental Information.
