# Supplementary material for: Factors associated with sufficient knowledge of antibiotics and antimicrobial resistance in the Japanese general population
Source: Sci Rep. 2020 Feb 26;10:3502. doi: 10.1038/s41598-020-60444-1 (PMC7044168; doi:10.1038/s41598-020-60444-1)
Supplement: Supplementary file 1 — Supplemental Information. [file 41598_2020_60444_MOESM1_ESM.pdf]

Supplementary Digital Content for: Factors associated with sufficient knowledge of antibiotics and antimicrobial resistance in the Japanese general population

Shinya Tsuzuki*[1,2], Niina Fujitsuka[3], Keisuke Horiuchi[3], Shinpei Ijichi[4], Yoshiaki Gu[1], Yumiko Fujitomo[1], Rie Takahashi[1], and Norio Ohmagari[1,5]

[1]AMR Clinical Reference Center, National Center for Global Health and Medicine, Tokyo, Japan

[2]Faculty of Medicine and Health Sciences, University of Antwerp, Antwerp, Belgium

[3]NTT Data, Tokyo, Japan

[4]DataRobot Japan, Tokyo, Japan

[5]Disease Control and Prevention Center, National Center for Global Health and Medicine, Tokyo, Japan

*Corresponding author

Supplementary file S1

English translation of the original questionnaire

Survey on antimicrobials (antibiotics)

Thank you for participating in our survey.

This survey is endorsed by the "National Action Plan on Antimicrobial Resistance (AMR)" and we request your participation.<br>The survey includes questions about your intake of medication (specifically antimicrobials (antibiotics)*).<br>We appreciate your understanding and participation in this survey.<br><br>*"Antimicrobials (antibiotics)" are prescribed drugs to treat infections, and the term includes drugs such as antibiotics in this survey.<br>Its medicated form may be pills, powder, or syrup. Please keep all antimicrobials (antibiotics) in mind when answering questions in this survey.<br><br>This survey may ask questions about yourself and your family.

If you consent to participation, please click the [Start] button and begin the survey.

[Start]

Caution:

Please do not click [Back] in your browser while answering the survey.

-----<page break>--

Q1	What is your gender?

1 Male
2 Female

Q2	What is your age?

1 [ ] years old <size=10><must><min=0><max=100>

-----<page break>--

Q3	What is your occupational status?

1 Full time (general)
2 Full time (managerial)
3 Non-full time (part-time / contract / temporary)
4 Dispatched worker
5 Self-employed / family business employee / side job
6 Other; please specify: <must><size=20><len<=50>
7 Do not work (retired / housewife / househusband / student / etc.)

-----<page break>--

[Condition]
Selected any answer between "1 Full time…" to "6 Other…" for Q3

Q4	What industry do you work in?

1 Agriculture / Fishing / Forestry / Mining	14 Transportation / Warehouse / Logistics-related
2 Civil engineering / Construction / Real estate / Building services	15 Electricity / Gas / Heat Supply / Water
3 Beverages	16 Telecommunications
4 Food / Food processing/ Health foods	17 Software, information processing, other information services
5 Cosmetics / Haircare	18 Mass media / Advertising, Newspaper / Broadcasting
6 Daily goods / Toiletries	19 Market survey / Think tank
7 Tobacco	20 Finance / Insurance (bank / security / insurance / wages, etc.)
8 Pharmaceutical	21 Restaurant / Hospitality services
9 Home appliances	22 Other services
10 Automotive (cars / motorcycles / etc.)	23 Education / Learning-related
11 Other manufacturing	24 Health care
12 Department store / Grocery store	25 Welfare
13 Convenience store / Other retail	26 Other

-----<page break>--

Q5	What municipality (prefecture and city / village / town) do you live in?

(Please specify)

Open selection page   Clear

1   <size=24>

2 [ ] <size=45>

Q5_num	Enter question here.<br>[This question will not be displayed on the screen when the answer is being input on the monitor]

1 [ ] <size=10>

2 [ ] <size=10>

-----<page break>--

Q6	What is your final degree?

1 Middle school graduate
2 High school graduate
3 Two-year college / Vocational college degree / Technical college graduate
4 University graduate
5 Graduate school graduate
6 Other
7 Rather not answer

-----<page break>--

Q7	How often do you use the internet?

1 Every day
2 Sometimes
3 Rarely

-----<page break>--

The next question is about "antimicrobials (antibiotics)"* you have taken in the past.

*"Antimicrobials (antibiotics)" are prescribed drugs to treat infections, and the term includes drugs such as antibiotics in this survey.

Its medicated form may be pills, powder, or syrup. Please keep all antimicrobials (antibiotics) in mind when answering questions in this survey.

Q8	Did you take any "antimicrobials (antibiotics)" within the past year?

1 Yes

2 No

-----<page break>--

[Condition]
Selected “1 Yes” for Q8

The following question is for those who have answered they have taken antimicrobials (antibiotics) in the past year.

Q9	Where did you obtain the antimicrobials (antibiotics) that you have most recently taken?

1 Prescribed at a hospital
2 Prescribed by a doctor at a clinic
3 Took leftover medicine from a previous prescription
4 Took non-prescribed medicine (nonprescription, over the counter)
5 Took medicine not prescribed anywhere (bought online, etc.)
6 I don’t really remember
7 I don’t know

-----<page break>--

[Condition]
Selected “1 Yes” for Q8

Q10	What was your reason for taking antimicrobials (antibiotics)?

1 Pneumonia
2 Bronchitis
3 Nasopharyngitis (inflammation of the nasal cavity and mucous membrane of the pharynx)
4 Influenza
5 Cold
6 Sore throat
7 Cough
8 Fever
9 Headache
10 Diarrhea
11 Urinary tract infection (UTI)
12 Skin infection or wound infection
13 Other
14 I don’t know <ex>

-----<page break>--

Q11	What are your thoughts on the following statements?

		1 Correct	2 Wrong	3 I don't know
1 Antimicrobials (antibiotics) kill viruses	→	○	○	○
2 Antimicrobials (antibiotics) are effective for colds and the flu	→	○	○	○
3 Unnecessary use of an antimicrobial (antibiotics) leads to that drug losing its effectiveness in the future	→	○	○	○
4 Antimicrobials (antibiotics) are bound to have side effects (such as diarrhea)	→	○	○	○
5 When prescribed antimicrobials (antibiotics), the entire prescribed amount must be taken	→	○	○	○

-----<page break>--

What are your thoughts on the following statements?

Q12	Everyone should be careful not to abuse antimicrobials (antibiotics) so that the next generation may benefit from the effects of antimicrobials (antibiotics).

1 Agree
2 Somewhat agree
3 Slightly disagree
4 Disagree
5 I don't know

-----<page break>--

Q13	In the past year, did you have a chance to become aware of how "antimicrobials (antibiotics) should not be taken unnecessarily (i.e., they should not be taken for colds or influenza, etc.)"?

1 Yes
2 I already knew
3 No

-----<page break>--

[Condition]
Selected "1 Yes" or "2 I already knew" for Q13

Q14	Where did you first learn that "antimicrobials (antibiotics) should not be taken

	unnecessarily"?

1 A doctor told me
2 A pharmacist told me
3 Another healthcare provider (nurse, physical therapist, etc.) told me
4 A family member or a friend told me
5 I learned from a television advertisement
6 I learned from a brochure (leaflet) or a poster
7 I learned from a newspaper or television news
8 I heard on the news
9 I learned online
10 Other
11 I don't know

-----<page break>---

[Condition]
Selected "1 Yes" or "2 I already knew" for Q13

Q15	Did your thoughts on antimicrobials (antibiotics) change after gaining knowledge that "antimicrobials (antibiotics) should not be taken unnecessarily"?

1 Yes
2 No
3 I don't know

-----<page break>---

[Condition]
Selected "1 Yes" for Q15

Q16	How specifically did your thoughts on antimicrobials (antibiotics) change after gaining knowledge that "antimicrobials (antibiotics) should not be taken unnecessarily"?

1 I now always consult a doctor when I feel that I need antimicrobials (antibiotics)
2 I no longer take antimicrobials (antibiotics) using my own judgment
3 I no longer take antimicrobials (antibiotics) that are not prescribed by a doctor
4 I no longer keep leftover antimicrobials (antibiotics) (in case I get sick again)
5 Other
6 Nothing has changed <ex>
7 I don't know <ex>

-----<page break>---

Q17	When attempting to gain proper information on antimicrobials (antibiotics), what is your source of information? Please select up to 3.

1 Doctor	12 AMR Clinical Reference Center's website
2 Nurse	13 Other wellness-related websites
3 Pharmacist	14 AMR Clinical Reference Center's SNS and videos
4 Hospital	15 Other SNS or video streaming services
5 Other healthcare facility (clinic, dental clinic, etc.)	16 TV and radio
6 Pharmacy	17 Health and medical magazines such as *Katei No Igaku*
7 Family or friends	18 Newspapers or general magazines
8 Personal or corporate websites	19 Wellness-related magazines
9 Municipal, public health center, medical association websites	20 Other (heard somewhere, etc.)
10 Government or Ministry of Health, Labour and Welfare websites	21 I don't intend to gain information on antimicrobials (antibiotics) <ex>
11 National Institute of Infectious Diseases' website	22 I don't know <ex>

-----<page break>---

Q18	Have you heard of antimicrobial resistance (AMR)?

1 Yes
2 No

-----<page break>---

Q19	What are your thoughts on the following statements?

		1 Correct	2 Wrong	3 I don't know
1 Antimicrobial resistance is a condition where a person physically becomes less likely to gain benefits of antimicrobials (antibiotics) or develops immunity or resistance to antimicrobials (antibiotics)	→	○	○	○
2 Antimicrobial resistance is a condition where bacteria become less susceptible to antimicrobials (antibiotics) or develop resistance to antimicrobials (antibiotics)	→	○	○	○

-----<page break>---

Q20	What do you think is the cause of antimicrobial resistance? Please select all that you think applies.

1 Unnecessary use of antimicrobials (antibiotics)
2 Excessive use of antimicrobials (antibiotics)
3 Lack of proper measures at the hospital (inconsistent hand-washing practices, not checking for antimicrobial resistance occurrences, etc.)
4 Stop taking antimicrobials (antibiotics) during treatment
5 Other
6 I don't know <ex>

-----<page break>---

Q21	Does the following statement apply to you?

		1 Yes	2 No
1 I keep antimicrobials (antibiotics) at home	→	○	○
2 I have suspended or adjusted dosage of antimicrobials (antibiotics) during treatment on my own judgment	→	○	○

-----<page break>---

[Condition]
Selected "1 Yes" for Q21 "I keep antimicrobials…"

-----<page break>---

Q22	This is a question for those who keep antimicrobials (antibiotics) at home.

		1 Yes	2 No
1 Have you used the antimicrobials (antibiotics) that you keep at home?	→	○	○
2 Have you given the antimicrobials (antibiotics) you keep at home to friends and family, and have they used them?	→	○	○

-----<page break>---

Q23	Does the following statement apply to you?

		1 Yes	2 No
1 I was administered the flu vaccine between fall 2018 and winter 2019	→	○	○
2 I caught a cold or the flu in the past year	→	○	○

-----<page break>---

[Condition]
Selected "1 Yes" for Q23 "2 I caught a cold or the flue…"

This question is for those who caught a cold or the flue in the past year.

Q24	Did you visit a health-care facility when you caught a cold or the flu?

1 Yes
2 No

-----<page break>--

[Condition]
Selected “1 Yes” for Q24

Q25	When did you visit a health-care facility after experiencing symptoms?

1 First day
2 Second day
3 Third day
4 Fourth day
5 Fifth day or later

-----<page break>--

Q26	Do the following statements apply to you?

		1 Yes	2 No
1 I have asked my doctor to prescribe antimicrobials (antibiotics)	→	○	○
2 I think doctors who prescribe antimicrobials (antibiotics) to treat colds are good doctors	→	○	○

-----<page break>--

Supplementary file S2

APPENDIX: Description of selected models

Model 1. Elastic-net blender (Knowledge of antibiotics, Data 1)

Model 2. Regularized logistic regression (Knowledge of antibiotics, Data 2)

Model 3. Regularized logistic regression (Knowledge of antibiotics, Data 3)

Model 4. Elastic-net blender (Knowledge of AMR, Data 1)

Model 5. Elastic-net blender (Knowledge of AMR, Data 2)

Model 6. Regularized logistic regression (Knowledge of AMR, Data 3)

Model 7. eXtreme gradient boosted tree classifier (Behavioural change, Data 1)

Model 8. Regularized logistic regression (Behavioural change, Data 2)

Model 9. Gradient boosted tree classifier (Behavioural change, Data 3)

Model 1. Elastic-net blender (Knowledge of antibiotics, Data 1)

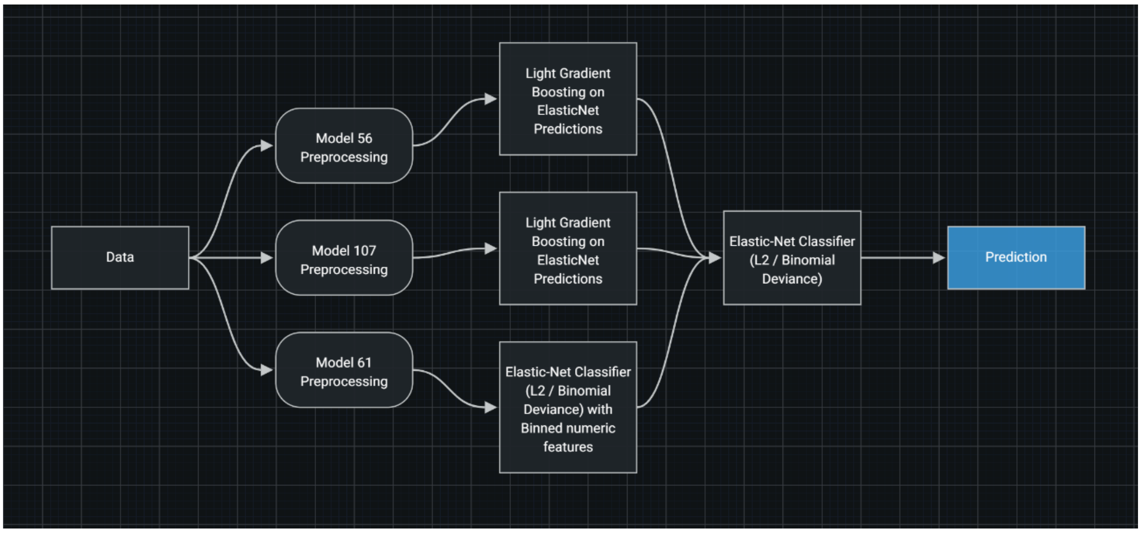

This ensemble learning model takes predictions from several input models and averages them in a meta-model. The final prediction is estimated by the elastic-net classifier.

The elastic-net is an extension of logistic regression where the optimizer attempts to find a parsimonious model. A simpler model is defined as one with coefficients with smaller absolute values and fewer non-zero coefficients. This can accommodate co-linear variables and produce models that are less prone to overfitting and are more generalizable to new data. This "preference for simpler models" is formally defined as "regularization," and the degree of regularization for non-zero coefficients as well as the absolute value of the coefficients are the two major meta parameters

Elastic-net is a linear regression model trained with L1 and L2 priors for regularization. This combination allows for learning a sparse model where few of the weights are non-zero, such as Lasso, while still maintaining the regularization properties

of Ridge. Elastic-net is useful when multiple features are correlated with one another. Lasso is likely to pick one of these at random, while elastic-net is likely to pick both. A practical advantage of this trade-off between Lasso and Ridge is that elastic-net can inherit the stability of Ridge under rotation.

Model 2. Regularized logistic regression (Knowledge of antibiotics, Data 2)

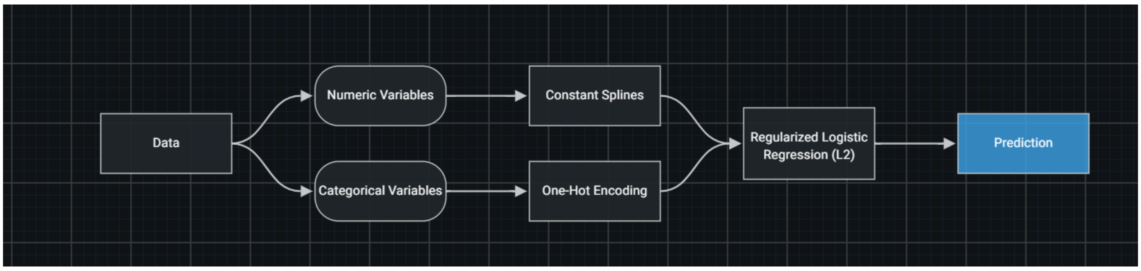

Logistic is a class of generalized linear models that uses the binomial distribution to fit regression models to a binary (0/1) response variable. It provides a good baseline by which to judge the performance of other classifiers. Logistic regression tends to produce well-calibrated classification probabilities without the need for post-processing.

Model 3. Regularized logistic regression (Knowledge of antibiotics, Data 3)

This model has the same structure as Model 2.

Model 4. Elastic-net blender (Knowledge of AMR, Data 1)

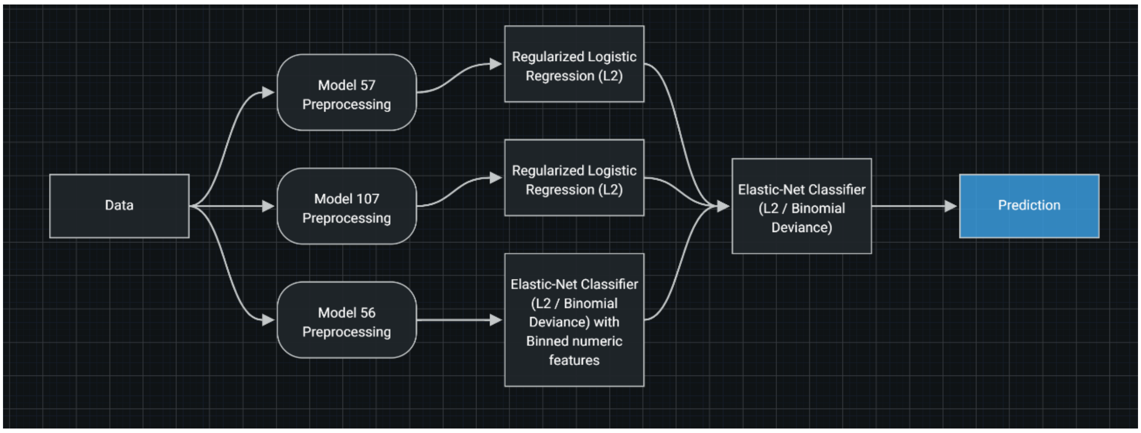

This is also an ensemble learning model with different preprocessing steps from those of Model 1. The final prediction was estimated using an elastic-net classifier.

Model 5. Elastic-net blender (Knowledge of AMR, Data 2)

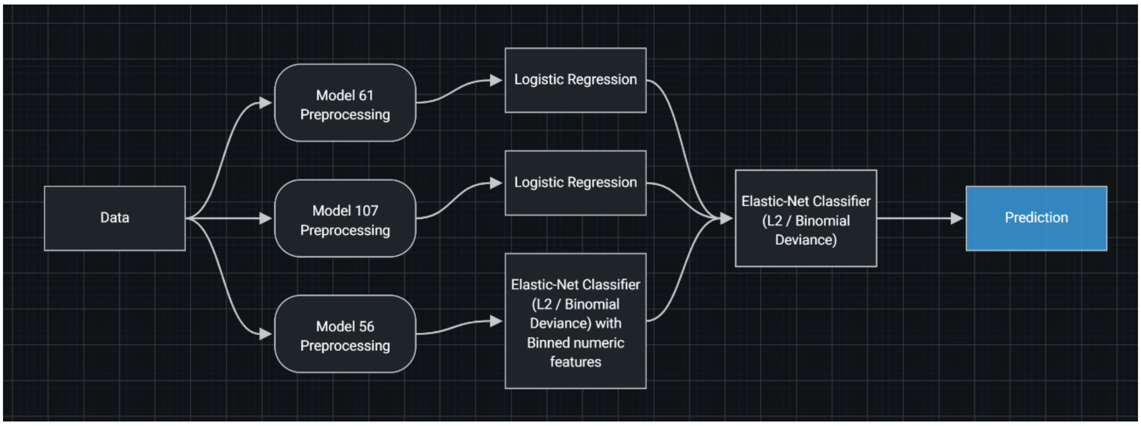

This is another ensemble learning model with different preprocessing steps from those of Models 1 and 4. The final prediction was estimated using an elastic-net classifier.

Model 6. Regularized logistic regression (Knowledge of AMR, Data 3)

This model has the same structure as Models 2 and 3.

Model 7. eXtreme gradient boosted tree classifier (Behavioural change, Data 1)

Gradient boosting machines (or generalized boosted models) are a cutting-edge algorithm for fitting extremely accurate predictive models. GBMs require very little preprocessing, elegantly handle missing data, strike a good balance between bias and variance and are typically able to find complicated interaction terms, which makes them a useful tool for predictive models.

GBMs are a generalization of Freund and Schapire's AdaBoost algorithm (1995) for handling arbitrary loss functions. They are very similar in concept to random forests, in that they fit individual decision trees to random re-samples of input data, where each tree sees a bootstrap sample of the rows of a dataset and N arbitrarily chosen columns (where N is a configural parameter of the model). GBMs differ from random forests in a single major aspect: rather than fitting the trees in parallel, each successive tree is fitted to the residual errors from all previous trees combined. This is advantageous, as the model focuses each iteration on the examples that are most difficult to predict (and therefore

most useful to get correct).

Extreme gradient boosting is a very efficient, parallel version of GBM. The base algorithm is very similar to GBM in R or in Python, but it has been heavily optimized and tweaked for faster runtimes and higher predictive accuracy. It uses logistic loss. The best number of trees is selected after the model has been fitted.

Model 8. Regularized logistic regression (Behavioural change, Data 2)

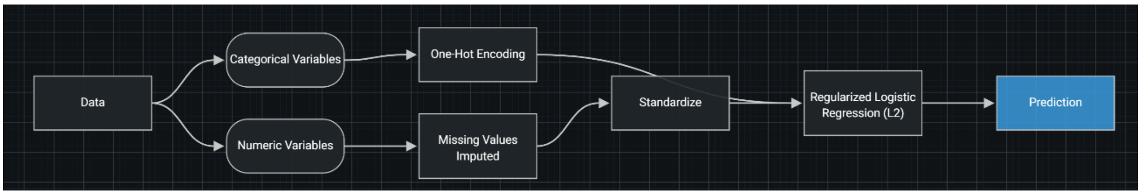

This model has a structure similar to those of Models 2, 3 and 6 but differs in the preprocessing of numeric variables.

Model 9. Gradient boosted trees classifier (Behavioural change, Data 3)

This model is based on GBMs, as explained in the description of Model 7.

Supplementary file S3

Sensitivity analysis: a multi-class classification approach

We conducted sensitivity analyses by multi-class classification approach. We classified all participants in accordance with the number of correct answers for each question (knowledge of antibiotics, knowledge of AMR, and behavioural change).

Table S1. Best fitting model for each dataset

	Data 1	Data 2	Data 3
**Has sufficient knowledge of antibiotics**			
Model	TensorFlow blender	AVG blender	ENET blender
AUC*	0.580	0.584	0.620
**Has sufficient knowledge of AMR**			
Model	ENET blender	AVG blender	AVG blender
AUC*	0.620	0.614	0.616
**Behavioural change after obtaining knowledge of AMR**			
Model	AVG blender	AVG blender	AVG blender
AUC*	0.619	0.618	0.618

AMR; antimicrobial resistance, ENET; elastic-net, AUC; Area under the curve

*AUC value from Five-fold validation with all the subsets.

Table S2. Common variables associated* with the dependent variables in all datasets

	Data 1	Data 2	Data 3
**Has sufficient knowledge of antibiotics**			
Education level	0.96	1.0	1.0
Use of primary information	0.84	0.92	0.30
Frequency of internet use	0.83	0.73	0.28
**Has sufficient knowledge of AMR**			
Education level	1.0	1.0	1.0
Use of primary information	0.45	0.78	0.60
Frequency of internet use	0.38	0.63	0.55
City size	0.12	0.35	0.23
**Behavioural change after obtaining knowledge of AMR**			
Has had opportunities to obtain knowledge about AMR in the past year	1.0	1.0	0.98
Everyone should limit antibiotic abuse for the next generation	0.95	0.83	1.0
Life expectancy of the prefecture you live	0.51	0.79	0.46
Use of primary information	0.53	0.60	0.56
Age group	0.42	0.53	0.44
Have quit antibiotic treatment before course completion	0.44	0.37	0.39
Gender	0.40	0.45	0.32
Education level	0.25	0.31	0.30
City size	0.22	0.29	0.25
Housewife/husband	0.20	0.29	0.21

Numbers represent permutation importance. AMR; antimicrobial resistance

*Variables ranked among the top 10 in permutation importance in all three datasets.
